# Afterimage duration differs for migraine with or without aura

**DOI:** 10.1111/head.14934

**Published:** 2025-03-28

**Authors:** Florian Rimmele, Julia Teuber, Britta Müller, Simeon Giesen, Johannes Drescher, Jörg Scheidt, Uwe Walter, Peter Kropp, Tim P. Jürgens

**Affiliations:** ^1^ Department of Neurology, Headache Centre North‐East Rostock University Medical Centre Rostock Germany; ^2^ Institute of Medical Psychology and Medical Sociology Rostock University Medical Centre Rostock Germany; ^3^ Institute for Information Systems Hof University Hof Germany; ^4^ Department of Neurology, Headache Centre North‐East KMG Hospital Güstrow Güstrow Germany

**Keywords:** afterimage, headache, migraine, palinopsia

## Abstract

**Background:**

It is controversial to what extent afterimages, as distinct visual phenomena, are altered in patients with migraine and whether they have a specific role in migraine pathophysiology.

**Objective:**

The aim of this cross‐sectional study was to investigate the duration of afterimages in patients with migraine, migraine with aura (MwA), and migraine without aura (MwoA), compared to healthy controls (HCs).

**Methods:**

Adults with migraine, MwA, and MwoA, diagnosed according to The International Classification of Headache Disorders, third edition criteria and HCs without relevant headache history were included. Initially, factors affecting the experimental setting of testing afterimage latency were determined. Then, afterimage duration was measured in the two study groups (MwA and MwoA) and the HC group. Patient characteristics, intraocular pressure, and relevant comorbid conditions, as well as scales on depressive symptoms (nine‐item Patient Health Questionnaire) and headache‐specific psychosocial impairment (six‐item Headache Impact Test) were recorded. Lastly, the role of different stimulus colors, as well as habituation effects after repeated stimulation, were investigated.

**Results:**

The main study included 174 participants (40 with MwA, 53 with MwoA, and 81 HCs). The duration of the afterimage in patients with MwA was significantly longer than in HCs, at a mean (standard error of the mean [SEM]) of 12.6 (2.6) versus 5.5 ( 0.3) s (*p* = 0.035), while there was no significant difference between patients with MwoA (mean [SEM] 7.7 [1.6] s; *p* = 0.510) and HCs. There was also no significant effect of stimulus color on afterimage latency (mean [SEM] red: 8.9 [1.2] s and black: 8.4 [1.2] s).

**Conclusion:**

We found significantly longer afterimage duration in patients with MwA compared to both HCs and patients with MwoA. Furthermore, partially selective stimulation of retinal rods and cones by different stimulus colors had no effect on afterimage duration suggesting a relevant subcortical and/or cortical modulation in migraine aura with increased excitability.

## Plain Language Summary

The visual system plays an important role in the pathophysiology of migraine, and afterimages (the perception of an image after visual stimulation has ceased) can be used to investigate this system. In this study, we investigated afterimage duration in a total of 174 adults (patients with migraine with and without aura, as well as healthy control participants) and found a longer afterimage duration in migraine with aura compared to the other groups. Stimulation with a black or red stimulus had no effect on afterimage duration, suggesting that there may be a different pathophysiology between migraine without and with aura and a modulation of afterimages at the subcortical and/or cortical level.

**Abbreviations**
ANOVA: analysis of variance
CSD: cortical spreading depression
HC: healthy control
HIT‐6: six‐item Headache Impact Test
LGN: lateral geniculate nucleus
MwA: migraine with aura
MwoA: migraine without aura
PHQ‐9: nine‐item Patient Health Questionnaire
SD: standard deviation
SEM: standard error of the mean
TMS: transcranial magnetic stimulation
VEP: visual evoked potentials

## INTRODUCTION

The visual system plays an important role in the pathophysiology of both migraine with aura (MwA) and migraine without aura (MwoA).^1^ Frequently reported alterations include visual aura and photophobia during an attack, while others receive less attention, such as afterimages. Hypersensitivity to visual stimuli is a typical characteristic of migraine, most likely due to an altered excitability of the visual cortex shown in various studies using electrophysiological approaches.^2^, ^3^ Afterimages are a physiological phenomenon in which images are experienced beyond presentation of the visual stimulus. Perception of the afterimage depends on stimulus intensity, stimulus contrast, fixation time, and adaptation processes. The image can be seen in the complementary color (negative afterimage) when looking at a bright background or in the same color (positive afterimage) when looking at a dark background.^4^, ^5^ Afterimage duration is likely to be influenced by excitatory and inhibitory states of visual cortex and postretinal relay stations. Therefore, afterimage duration may be a clinical marker of sensitization and altered habituation of sensory neurons.^6^ In previous studies, the terms palinopsia and afterimage were sometimes used synonymously, and different study paradigms and cohorts were used, resulting in different and sometimes contradictory results.^4^, ^7^ The results of recent studies are controversial as to whether or not the duration of afterimages are longer or shorter in patients with migraine compared to healthy controls (HCs), and the inherent consequences for migraine pathophysiology.^6^, ^8^, ^9^, ^10^ Most studies have not distinguished between MwA and MwoA, nor have they taken into account the blink effect, which can lead to reactivation of afterimages resulting in a prolongation of afterimage latency.^4^

The aim of this study was to investigate afterimage duration using a standardized experimental paradigm in patients with MwA and patients with MwoA versus HCs. The primary hypothesis is that patients with MwA have a longer afterimage duration due to increased cortical excitability. The secondary hypotheses are that the afterimage duration of the black stimulus does not differ significantly from that of the red stimulus, as the afterimages are modulated at the subcortical and/or cortical, but not the retinal level. Additionally, it is hypothesized that patients with migraine show constant result of the afterimage duration after repeated testing compared to HCs as an expression of a habituation deficit.

## METHODS

**FIGURE 1:**
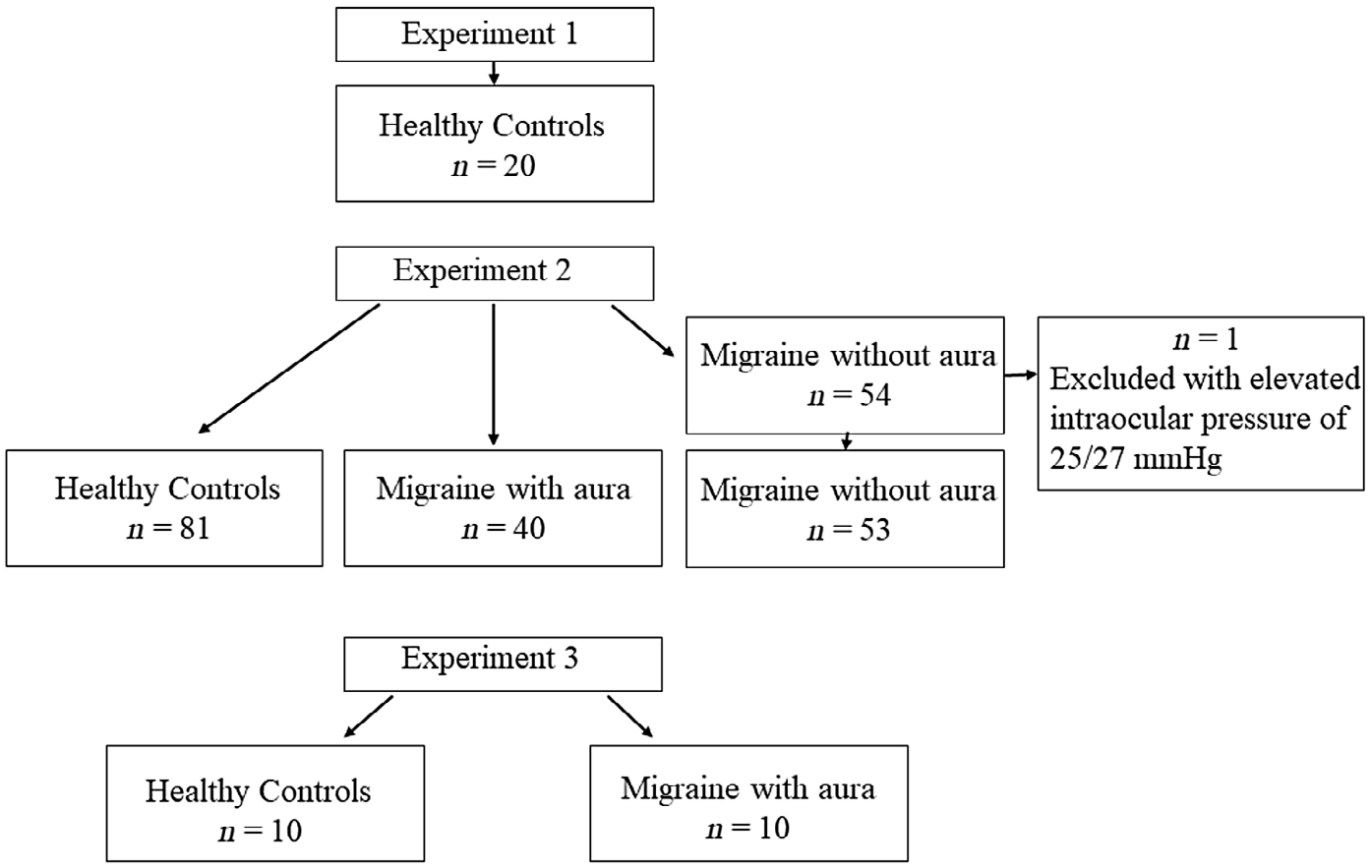
Flow chart.

This cross‐sectional study was divided into three parts (see flow chart in Figure 1). In the first experiment, the influence of external factors on afterimage duration, such as distance between participant and screen, screen brightness, and ambient brightness was investigated. In the second experiment, the duration of afterimage perception was measured in HCs and patients with MwoA or MwA. In the last experiment, the influence of a colored stimulus versus an achromatic stimulus was investigated. To assess habituation, 10 measurements of the afterimage latency were compared for both chromatic and achromatic stimuli for the same participants.

### Participants

The present study included participants aged ≥18 years with a medical diagnosis of MwA or MwoA according to the International Classification of Headache Disorders, third edition criteria.^11^ The HCs were not allowed to have a history of migraine or trigeminal autonomic cephalalgia, although tension‐type headache on <2 headache days/month was acceptable. The participants for Experiment 1 were recruited from students at the University of Rostock without migraine or other concomitant conditions. The participants with MwA or MwoA for Experiment 2 were recruited at the Headache Center North‐East of the Rostock University Medical Center, while employees and students at the Rostock University Medical Center served as HCs. Both patients with migraine and HCs for Experiment 3 were recruited among staff and students at the Rostock University Medical Center. The data were collected between October 2020 and February 2021. All participants were recruited for this specific purpose only and their data were not already published elsewhere, nor did they receive any compensation. All study participants were provided with written information about the study procedure prior to inclusion in the study and gave their written informed consent before participating in the study. The research was conducted in accordance with the declaration of Helsinki and approved by the Ethics committee of the Rostock University Medical Center (A2017‐0187).

### Measures

Afterimage duration was measured using a fixed experimental set‐up consisting of a tablet computer (iPad Pro™, Apple Inc., Cupertino, CA, USA) with a diagonal of 32.77 cm (12.9 inches) and a custom‐made spacer and wooden chin rest. The distance between the screen and the chin rest studied varied in Experiment 1 but was fixed at 50 cm in the consecutive experiments, while the viewing angle was fixed throughout all experiments. For stimulus presentation and measurement of the afterimage duration, a customized iOS application was used. Participants were instructed to fixate on the high‐contrast stimulus, consisting of a black circle with a black fixation cross at its center for 30 s without blinking. This was followed by a white screen with only the fixation cross, on which the afterimage would be perceived as a grayish circle.

In the first experiment, distance between participant and tablet (30, 40, 50 cm), screen brightness (0%, 50%, 100%), and ambient brightness (differing between lights on and off in the examination room) were tested. All measurements were done in a random order at intervals of 60 s.

In the second experiment, afterimage duration was measured at 50 cm and at a screen brightness of 50%, based on the results from Experiment 1. The participants were asked not to blink during the testing of the afterimage to avoid afterimage reactivation.^4^ Participants were then asked to blink after the disappearance of the first afterimage. If the afterimage could be reactivated by blinking, a second latency was measured. In the last experiment, afterimage duration was tested as in Experiment 2, except that stimulus color varied between red (wavelength 700 nm) and black (standard color). Two stimuli were given randomly with a pause of 120 s and 10 measurements were done repeatedly. After a wash‐out period of at least 24 h, testing with the second color was repeated.

Participants with a diagnosis of migraine had their medical history recorded, with a focus on ophthalmologic conditions, presence of a visual aid, and medication. HCs were screened in a similar fashion. Participants with migraine were asked to present their headache diaries, while participants in the HC group were asked about the frequency of any non‐migraine headache.

To assess depressive symptoms, the nine‐item Patient Health Questionnaire (PHQ‐9)^12^ was used, and to assess headache‐specific psychosocial status, the six‐item Headache Impact Test (HIT‐6) questionnaire^13^ responses were collected. Participants’ intraocular pressure was determined using an i‐Care ic100 Tonometer™ (icare Finland OY, Vantaa, Finland).^14^ Normative values for intraocular pressure ranged between 10 and 21 mmHg.

### Analyses

This primary analysis of the dataset was planned a priori. No statistical power calculation was conducted prior to the study. The sample size was based on our previous experience with a similar design. For statistical analysis, the IBM Statistical Package for the Social Sciences (SPSS™) was used (IBM Corp., Armonk, NY, USA). The main variables were analyzed using descriptive statistics (median, mean, standard deviation [SD], percentage, and the standard error of the mean [SEM]). For Experiment 1, a repeated‐measures analysis of variance (ANOVA) was performed to evaluate the effect of the distance factors (30, 40, 50 cm), ambient brightness (light on, light off), and screen brightness (0%, 50%, 100%) on afterimage duration in HCs.

For Experiment 2, one‐way ANOVAs were used to evaluate effects on the factors group (HC, MwoA, MwA), and latency (first latency and second latency) on afterimage duration, PHQ‐9 score, and the HIT‐6 score. For Experiment 3, a repeated‐measures ANOVA with a Greenhouse–Geisser correction was performed to evaluate the effect of the factors time (10 measurements), stimulus (red and black), group (HC, MwA), and latency (first; second) on the afterimage duration. For the correlation of items with the first latency in Experiment 2 a Pearson's correlation was used, and Mann–Whitney *U* tests for ordinally scaled data. For participants who did not perceive an afterimage, both for the first latency and after reactivation with blinking (second latency), latencies were scored as zero. All hypothesis tests were two‐tailed and considered statistically significant at a *p* < 0.05.

## RESULTS

### Experiment 1

In the first experiment, 20 HCs without headache disorders were examined (10 females, 10 males; mean [SD] age 23.2 [0.9] years). Univariate analysis of variance showed no significant effect of distance (*p* = 0.378), screen brightness (*p* = 0.751), and ambient light (*p* = 0.679) on afterimage latencies. ANOVA for the factors: ambient illumination and distance (*p* = 0.252), screen brightness and distance (*p* = 0.291), screen brightness and ambient illumination (*p* = 0.291) on afterimage latency, as well as the combination of all three factors jointly (*p* = 0.534), revealed no significant effect (Data S1, Supporting Information S1).

### Experiment 2

**FIGURE 2:**
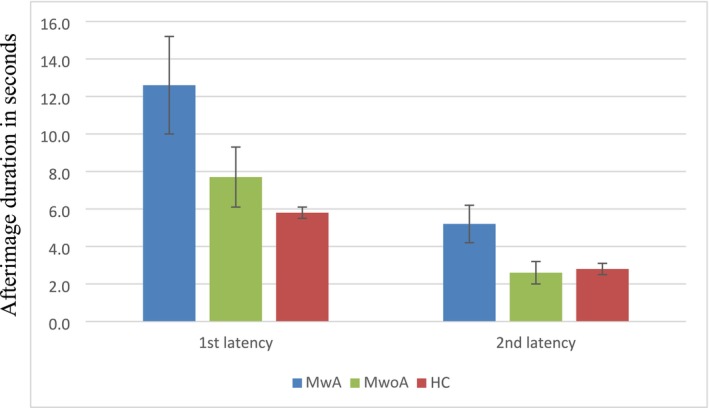
Afterimage duration from Experiment 2 in the groups: Migraine with aura (MwA), migraine without aura (MwoA), and health controls (HC). [Colour figure can be viewed at wileyonlinelibrary.com]

In Experiment 2, afterimage durations were measured in 40 patients with MwA (34 females; mean [SD] age 37.9 [1.9] years), 53 patients with MwoA (43 females; mean [SD] age 45.8 [1.9] years), and 81 HCs (67 females; mean [SD] age 40.6 [1.5] years). The afterimage duration was significantly longer in patients with MwA (mean [SEM] 12.6 [ 2.6] s) compared to HCs (mean [SEM] 5.8 [0.3] s; *p* = 0.035), both for the first (*p* = 0.035) and the second latency (mean [SEM] 5.2 [1.0] s; *p* = 0.002). There was no significant difference in patients with MwoA (mean [SEM] first latency: 7.7 [1.6] s; *p* = 0.510 and second latency: 2.6 [0.6] s; *p* = 0.800) compared to HCs; Figure 2. The duration of the afterimage was not significantly longer for the first latency (*p* = 0.250), but was for the second latency (*p* = 0.004) in MwA compared to MwoA (Figure 2). Of the HCs, 8.6% missed the first afterimage and 39.5% missed the second afterimage; of the patients with MwoA, 18.9% missed the first afterimage and 37.7% the second afterimage; and of the patients with MwA, 16.7% missed the first afterimage and 23.3% the second afterimage.

Further details of headache characteristics such as duration of illness and headache days/month are summarized in Table 1. There was no significant correlation of the individual headache characteristics with afterimage duration (Data S1, Supporting Information S2 and S3).

**TABLE 1:** Headache characteristics (Experiment 2).

Characteristic	Migraine without aura (*n* = 53)	Migraine with aura (*n* = 40)	Healthy controls (*n* = 81)
Duration of disease, years, mean (SD)	25.2 (3.0)	21.3 (1.7)	–
Migraine attack duration, h, mean (SD)	33.8 (3.9)	30.2 (4.5)	–
Duration of the aura, h, mean (SD)	–	1.02 (0.2)	–
Headache intensity, NRS score, mean (SD)	7.2 (0.4)	6.9 (0.4)	–
Headache days per month, mean (SD)	12.4 (1.6)	10.9 (1.1)	2 (0.4)^a^
Headache on the day of the examination, %	43.4	41.5	0.0
Headache intensity on examination day if headache is present, NRS score, mean (SD)	4.4 (0.7)	3.9 (0.4)	–
Days since the last migraine attack, mean (SD)	11.8 (7.5)	11.9 (3.9)	–

Abbreviations: NRS, numeric rating scale; SD, standard deviation.

^a^ The participants were examined by a doctor at the local headache center and the headache was rated as an occasional, non‐migraine headache.

In the PHQ‐9 sum score, patients with MwA had a mean (SD) score of 9.4 (1.2), patients with MwoA had a score of 7.2 (0.7), and HCs had a score of 3.8 (0.4). Depressive symptoms were found in 61.4% of patients with migraine, of whom 6.0% had severe depressive symptoms (four with MwA, one with MwoA), 7.0% had moderate depressive symptoms (three with MwA, three with MwoA), and 12.0% had mild depressive symptoms (five with MwA, five with MwoA). Two of the HCs had mild depressive symptoms. There was no correlation between afterimage duration and the PHQ‐9 score in any of the three groups (Data S1, Supporting Information S4).

For the HIT‐6, patients with MwA had a mean (SD) score of 64.2 (1.1), patients with MwoA had a score of 61.1 (0.9), and HCs had a score of 43.2 (1.2). This corresponds to the category “severe impact of headache” in the HIT‐6 for patients with MwA and MwoA and to the category “little or no impact of headache” for HCs. There was no association of afterimage duration with the HIT‐6 score for any of the three groups (Data S1, Supporting Information S4).

Frequency of use of visual aids, eye diseases, and the mean intraocular pressure are shown in Data S1, Supporting Information S5. There were no significant differences among the groups. One patient with MwoA had an elevated intraocular pressure of 25/27 mm Hg and was excluded from the study. The medications taken by the participants are listed in Data S1, Supporting Information S6.

### Experiment 3

**FIGURE 3:**
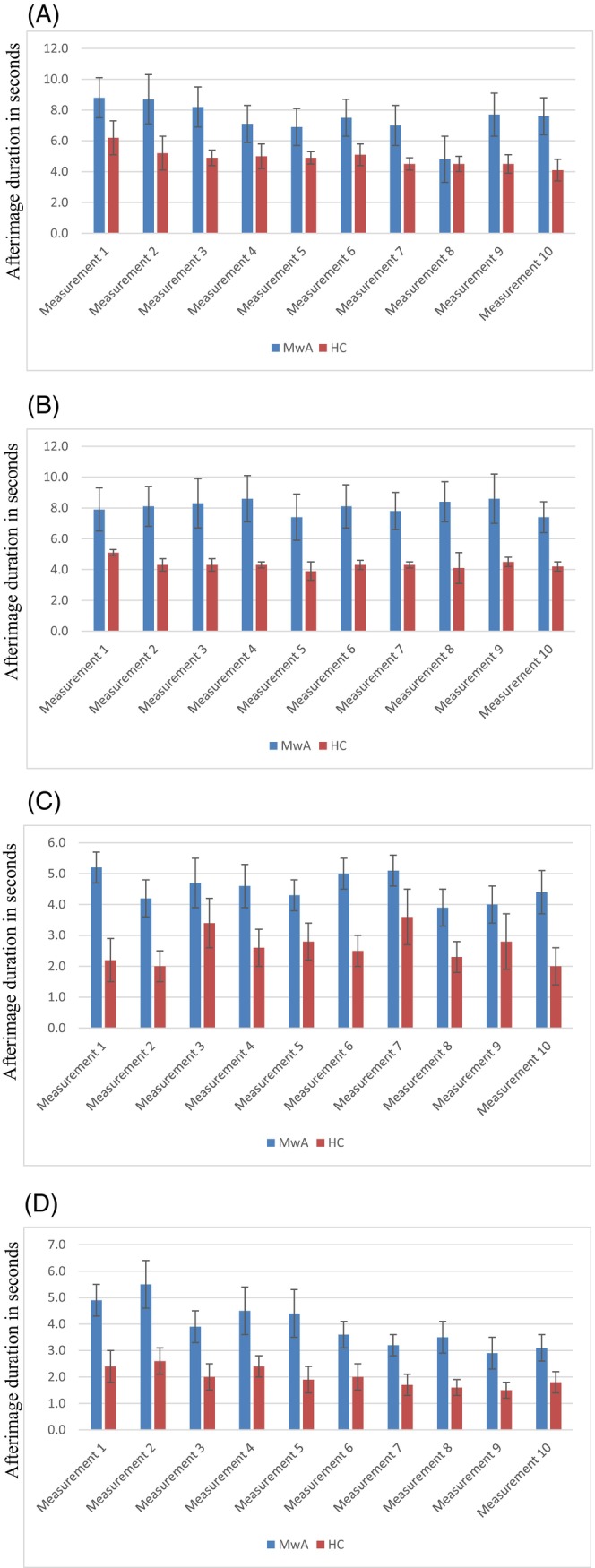
Afterimage duration from Experiment 3. (A) Series with red stimulus (first latency), (B) series with black stimulus (first latency), (C) series with red stimulus (second latency), (D) series with black stimulus (second latency). Migraine with aura (MwA), health controls (HC). [Colour figure can be viewed at wileyonlinelibrary.com]

Experiment 3 examined the duration of afterimages following the presentation of a black and a red stimulus in 10 patients with MwA (mean [SD] age 29.6 [2.7] years) and 10 HCs (mean [SD] age 25.6 [0.4] years). Again, a longer afterimage duration was found in patients with MwA (mean [SEM] 8.4 [1.2] s for black; 8.9 [1.2] s for red) compared to HCs (mean [SEM] 4.8 [0.4] s for black, *p* = 0.017; 5.6 [0.5] s for red, *p* = 0.030) following both black and red stimuli. There was no significant difference in afterimage duration between these groups when either a black or red stimulus was applied. A summary of the details pertaining to the headaches experienced by the participants and their characteristics are summarized in Data S1, Supporting Information S7. The measurement was repeated 10 times in order to investigate a potential habituation to the stimulus. Following the initial measurement of the first latency, no difference was observed between the groups (*p* = 0.087), whereas the subsequent measurements differed significantly. Thus, latencies were initially longer in patients with MwA from the second measurement onwards and only decreased over time, whereas latencies in HCs decreased from the second measurement onwards. The afterimage latencies are shown in Figure 3. As patients with MwA more commonly showed a habitual deficit to the stimulus compared to HCs, we performed a robust ANOVA of the repeated measures of the afterimage latencies. However, the four‐factor ANOVA revealed no significant influence of the measurement between the two groups.

## DISCUSSION

The results of this study indicate that the duration of afterimages was significantly longer in patients with MwA than in headache‐free participants (HCs). Conversely, no prolongation was observed in patients with MwoA. This implies differences in the pathophysiology of MwA and MwoA, although the precise physiological mechanisms responsible for changes in afterimage duration remain uncertain. The current literature is inconclusive regarding the question of whether afterimage duration is shorter or longer in patients with migraine than in HCs.^6^, ^8^, ^9^, ^10^ In a previous study, afterimage duration was unable to distinguish between MwA and MwoA, but demonstrated changes over the migraine cycle. The afterimage duration was found to be prolonged in ictal patients with migraine, while interictal patients with migraine exhibited a shortened afterimage duration.^9^ However, the study did not distinguish between MwA and MwoA, nor did it account for blinking in a systematic manner, as was done in our present study. This may have resulted in an artificial prolongation of afterimage duration.^15^ This finding of increased afterimage duration for MwA represents a new result.

What conclusions can be drawn regarding the pathophysiology of migraine considering the change in the afterimage? It is likely that the afterimage originates in the visual pathway at the retinal level and is modified at the subcortical level, in the lateral geniculate nucleus (LGN), and/or at the cortical level in the visual cortex.^9^, ^16^ The results of Experiment 3 provide compelling evidence against a purely retinal origin of afterimage duration without further modification, given that an at least partially targeted stimulation of retinal rods and cones did not affect afterimage duration. The thalamus, where the retinal ganglion cells are switched to the LGN and the receptive fields are retinotopically mapped, also plays an important role in central sensitization processes in migraine pathophysiology.^17^ In addition to the disparities in afterimage duration demonstrated here, differences in the pathophysiology of MwA and MwoA at this level have also been identified in studies employing disparate methodological approaches. For instance, functional magnetic resonance imaging showed that blood flow in the LGN is elevated in patients with MwA, but not in those with MwoA or HCs.^18^ It may therefore be possible that there is a pathophysiological difference between MwA and MwoA in the LGN that is responsible for the development of different afterimage durations. Additionally, our study revealed that a greater proportion of HCs than patients with MwA perceived no afterimage at all, which may suggest a differential filtering function of the thalamus in MwA. Disrupting the thalamocortical network in MwA may result in prolonged afterimage duration via a decreased lateral inhibition or a lowered preactivation at the cortical level.^19^, ^20^, ^21^, ^22^

The visual cortex represents the subsequent structure in the visual pathway where visual information can be modulated. Evidence from experiments with binocular rivalry indicates that afterimages may arise, at least in part, directly in the visual cortex.^23^ The cortical spreading depression model provides evidence that for the crucial role electrophysiological balance in visual cortex in MwA.^24^ Several studies have demonstrated cortical hyperexcitability in migraine,^25^, ^26^, ^27^ with some indicating also a distinction between MwoA and MwA, wherein cortical hyperexcitability is predominantly observed in MwA. Earlier studies employed visual evoked potentials^28^, ^29^ and transcranial magnetic stimulation^30^ for the purpose of assessing the visual system. Furthermore, studies of interictal brain activity in migraine using resting state functional magnetic resonance imaging have also provided evidence for cortical hyperexcitability in MwA rather than MwoA.^31^ It can therefore be posited that cortical hyperexcitability in MwA represents a modulating factor for the prolonged afterimage duration in MwA observed in our study. Cortical hyperexcitability may be associated with habituation deficits. In the present study (Experiment 3), patients with MwA more commonly showed a habitual deficit to the stimulus as compared to HCs, but this could not be confirmed as statistically significant in the subsequent ANOVA.

### Limitations

Several limitations of the present study should be considered. First, afterimage duration was not assessed longitudinally over the migraine cycle. As a result, specific differences in afterimage duration during the migraine attack versus the interictal phase could not be explicitly identified. The time elapsed since the patient's last migraine attack had no significant effect on afterimage duration, as observed in our study. Conversely, the inclusion of an additional recording of electrophysiological parameters for cortical hyperexcitability or habitation such as contingent negative variation would have been beneficial and should be considered in future studies. The relatively small group size in the individual experiments also represents a potential limitation, particularly in relation to the four‐factor analysis conducted in Experiment 3. However, the study's key strengths include the initial examination of the experimental setting for potentially influencing measurement variables (Experiment 1), as well as the avoidance of any blink‐related distortion of the afterimage duration. Furthermore, the results on afterimage duration were confirmed in a second cohort of patients (Experiment 3).

In conclusion, the results demonstrated a significantly longer afterimage duration in patients with MwA compared to both HCs and patients with MwoA. Neither chromatic nor achromatic stimuli had any effect on afterimage duration. It seems probable that the afterimages have a retinal origin with subcortical and/or cortical modulation. The differences between MwA and MwoA indicate that the pathophysiology may be different. Furthermore, the present study revealed that patients with MwA more commonly showed a habitual deficit to the stimulus as compared to HCs, suggesting that cortical hyperexcitability is a modulating factor of afterimages in MwA.

## AUTHOR CONTRIBUTIONS

**Florian Rimmele:** Formal analysis; investigation; methodology; writing – original draft; writing – review and editing. **Julia Teuber:** Data curation; formal analysis; investigation; writing – review and editing. **Britta Müller:** Formal analysis; methodology; writing – review and editing. **Simeon Giesen:** Formal analysis; writing – review and editing. **Johannes Drescher:** Software; writing – review and editing. **Jörg Scheidt:** Software; writing – review and editing. **Uwe Walter:** Formal analysis; writing – review and editing. **Peter Kropp:** Formal analysis; writing – review and editing. **Tim P. Jürgens:** Conceptualization; formal analysis; methodology; writing – review and editing.

## FUNDING INFORMATION

All authors are employees of the University Medical Center Rostock, Institute for Information Systems, Hof University or KMG Hospital Güstrow. Further funding of the article did not take place.

## CONFLICT OF INTEREST STATEMENT

**Florian Rimmele** served on advisory boards and/or as speaker for Allergan/Abbvie, Novartis, Teva, Ipsen, Lundbeck, Lilly, Hormosan. He has received royalties from Elsevier. **Uwe Walter** has received speaker honoraria and travel funds from Ipsen Pharma, Merz Pharma, Allergan, Bristol‐Myers Squibb, Daiichi Sankyo, Bayer Vital, Boehringer Ingelheim and Pfizer, and a research grant from Merz Pharma. He has received royalties from Thieme and Elsevier Press. He serves as editor of the *European Journal of Ultrasound*. **Peter Kropp** served on advisory boards and/or as speaker for Allergan, Novartis, Teva and Lilly. **Tim P. Jürgens** served on advisory boards and/or as speaker for Allergan, Abbvie, Autonomic Technologies, Desitin, Grünenthal, Hormosan, Novartis, Lilly, Lundbeck, Pfizer, Sanofi and Teva. **Julia Teuber, Britta Müller, Simeon Giesen, Johannes Drescher**, and **Jörg Scheidt** declare no competing interests.

## Supporting information


Data S1.

## Data Availability

All data generated or analyzed during this study are included in this published article or are available from the corresponding author on reasonable request.
