# Supplementary material for: Afterimage duration differs for migraine with or without aura
Source: Headache. 2025 Mar 28;65(5):756–63. doi: 10.1111/head.14934 (PMC12005615; doi:10.1111/head.14934)
Supplement: Supplementary file 1 — Data S1. [file HEAD-65-756-s001.docx]

Supplementary:

**Supp. 1** a.) Afterimage duration in seconds as a function of ambient brightness, screen brightness in % and distance from chin to iPad™ in cm.

|  | Distance/cm | Afterimage duration/sec |
| --- | --- | --- |
| Light on, 100% brightness | 50 | 7.7±0.7 |
|  | 40 | 7.7±0.8 |
|  | 30 | 7.6±0.9 |
| Light on, 50% brightness | 50 | 7.8±0.6 |
|  | 40 | 8.2±1.0 |
|  | 30 | 7.2±0.8 |
| Light on, 0% brightness | 50 | 7.8±0.9 |
|  | 40 | 7.2±0.8 |
|  | 30 | 6.6±0.7 |
| Light off, 100% brightness | 50 | 7.5±0.7 |
|  | 40 | 6.8±0.5 |
|  | 30 | 6.0±0.5 |
| Light off, 50% brightness | 50 | 8.5±0.8 |
|  | 40 | 6.9±0.6 |
|  | 30 | 6.8±0.8 |
| Light off, 0% brightness | 50 | 8.5±0.9 |
|  | 40 | 7.8±0.9 |
|  | 30 | 6.1±0.6 |

b.) Variance analysis showed no significant effect on afterimage latencies for:

| Distance | p=0.378 |
| --- | --- |
| Screen brightness | p=0.751 |
| Ambient light | p=0.679 |
| Ambient light* Distance | p=0.252 |
| Screen brightness* Distance | p=0.291 |
| Screen brightness* Ambient light | p=0.291 |
| Screen brightness* Ambient light* Distance | p=0.534 |

p=significance

**Supp. 2** Pearson correlation of the first afterimage duration; experiment 2.

|  | Migraine with aura (MA) | Migraine without aura (MoA) | Total migraine | All participants |
| --- | --- | --- | --- | --- |
| Headache days per month | r=0.01, p= 0.976 | r=0.06, p= 0.541 | r=0.04, p= 0.725 | r=0.09, p=0.875 |
| Headache on the day of the examination | r=0.02, p=0.885 | r=0.06, p=0.507 | r=0.01, p=0.896 | r=0.01, p=0.896 |
| Headache intensity (NRS^1^) on Examination day if headache is present | r=0.24, p=0.385 | r=0.07, p=0.750 | r=0.08, p=0.725 | r=0.09, p=0.632 |
| Days since the last migraine attack | r=0.13, p=0.461 | r=0.11, p=0.476 | r=0.01, p=0.389 | r=0.01, p=0.389 |

p=significance, r=Pearson correlation; ^1^ NRS: Numeric rating scale

**Supp. 3** Pearson correlation of the second afterimage duration; experiment 2.

|  | Migraine with aura (MA) | Migraine without aura (MoA) | Total migraine | All participants |
| --- | --- | --- | --- | --- |
| Headache days per month | r=0.01, p=0.769 | r=0.03, p=0.875 | r=0.04, p= 0.749 | r=0.06, p= 0.460 |
| Headache on the day of the examination | r=0.20, p=0.276 | r=0.09, p=0.624 | r=0.02, p=0.896 | r=0.08, p=0.517 |
| Headache intensity (NRS^1^) on Examination day if headache is present | r=0.08, p=0.385 | r=0.08, p=0.740 | r=0.08, p=0.632 | r=0.06, p=0.798 |
| Days since the last migraine attack | r=0.05, p=0.523 | r=0.05, p=0.775 | r=0.05, p=0.375 | r=0.05, p=0.731 |

p=significance, r=Pearson correlation; ^1^ NRS: Numeric rating scale

**Supp. 4** Correlation of afterimage duration with headache characteristics and scores of the HIT-6 and PHQ-9.

|  | Migraine with aura (MA) | Migraine without aura (MoA) | Healthy controls (HC) |
| --- | --- | --- | --- |
| Age (years) | p=0.792; r = -0.32 | p=0.408; r = -0.27 | p=0.699; r = -0.14 |
| missing | 1 | 1 | 1 |
|  |  |  |  |
| HIT-6 score | p=1.000, r = 0.03 | p=1.000, r = 0.01 | p=1.000, r = 0.07 |
| missing | 0 | 4 | 21 |
|  |  |  |  |
| PHQ-9 score | p=1.000, r = -0.08 | p=1.000, r = 0.01 | p=1.000, r = -0.01 |
| missing | 0 | 4 | 21 |
|  |  |  |  |
| Days since the last migraine attack | p=1.000, r = -0.08 | p=1.000, r = -0.11 |  |
| missing | 6 | 7 |  |
|  |  |  |  |
| Headache intensity (NRS) | p=1.000, r = 0.15 | p=1.000, r = -0.07 |  |
| missing | 0 | 0 |  |
|  |  |  |  |
| Migraine attack duration (hours) | p=1.000, r = 0.09 | p=1.000, r = -0.05 |  |
| missing | 0 | 2 |  |
|  |  |  |  |
| Headache days per month | p=1.000, r = -0.09 | p=1.000, r = -0.09 |  |
| missing | 0 | 2 |  |
|  |  |  |  |
| Duration of disease (years) | p=1.000, r = 0.01 | p=1.000, r = -0.01 | - |
| missing | 5 | 6 | - |
|  |  |  |  |
| Duration of the aura (hours) | p=1.000, r = 0.23 | - | - |
| missing | 2 | - | - |

p=Bonferroni-adjusted significance, r=Pearson product moment correlation coefficient; NRS: Numeric rating scale

**Supp. 5** Eye diseases, proportion of people wearing glasses and intraocular pressure (mmHg).

| Item | Migraine with aura (MA) | Migraine without aura (MoA) | Healthy controls (HC) |
| --- | --- | --- | --- |
| Glasses/ Contact lens | 55.0% | 68.0% | 74.0% |
| Eye disease | 6.9% | 18.0% | 6.7% |
| Intraocular pressure right | 11.7±1.0 | 12±1.1 | 10±1 |
| Intraocular pressure left | 11.7±0.9 | 12.7±1.4 | 8.5±2.5 |

**Supp. 6** Medication

| Medication | Migraine with aura (MA) | Migraine without aura (MoA) | Healthy controls (HC) |
| --- | --- | --- | --- |
| *Headache prophylaxis* | *44.0%* | *36.0%* | *0.0%* |
| Antidepressant | 33.0% | 15.0% | 0.0% |
| Antiseizure medication | 6.6% | 13.0% | 0.0% |
| β- Blocker | 6.6% | 20.0% | 6.0% |
|  |  |  |  |
| *Acute medication* | | | |
| Aspirin | 0.0% | 11.0% | 2.6% |
| Ibuprofen | 20.0% | 41.0% | 15.6% |
| Paracetamol | 6.6% | 5.6% | 0.0% |
| Metoclopramide | 6.6% | 7.5% | 0.0% |
| Novaminsulfon | 6.6% | 5.6% | 0.0% |
| Naproxen | 10.0% | 0.0% | 0.0% |
| Triptans | 30.0% | 33.0% | 0.0% |

**Supp. 7** Participants and headache characteristics (experiment 3)

|  | Migraine with aura (MA) | Healthy controls (HC) |  |
| --- | --- | --- | --- |
| Age (years) | 29.9±9.5 | 25.5±1.3 | p=0.194 |
| Sex (f/m) | 8/2 | 8/2 | p=0.921 |
| Headache years | 14.8±3.4 | 7.2±0.5 |  |
| Headache intensity (NRS) | 7.2±0.5 | - |  |
| Headache days per month | 3.3±0.8 | 1.5±0.7^1^ | p=0.187 |
| Headache on the day of the examination | 27.3% | 0.0% |  |
| Duration of the aura (min) | 62.7±25.4 | - |  |
| Days since the last migraine attack | 39.6±19.3 | - |  |
| Photophobia during a migraine attack | 90.9% | - |  |
| Phonophobia during a migraine attack | 45.5% | - |  |
| Glasses | 70.0% | 40.0% | p=0.370 |

NRS: Numeric rating scale; ±: standard deviation ^1^The participants were examined by a doctor at the local headache centre and the headache was rated as an occasional, non-migraine headache.
